# Very Long (> 48 hours) Shifts and Cardiovascular Strain in Firefighters: a Theoretical Framework

**DOI:** 10.1186/2052-4374-26-5

**Published:** 2014-03-06

**Authors:** BongKyoo Choi, Peter L Schnall, Marnie Dobson, Javier Garcia-Rivas, HyoungRyoul Kim, Frank Zaldivar, Leslie Israel, Dean Baker

**Affiliations:** 1 Center for Occupational and Environmental Health, University of California, Irvine, USA; 2 Department of Environmental Health, Korea University, Seoul, South Korea; 3 Center for Social Epidemiology, Marina Del Rey, California, USA; 4 Center for Occupational and Environmental Health, The Catholic University of Korea, Seoul, South Korea; 5 Department of Pediatrics, University of California, Irvine, USA

**Keywords:** 24-hr shift, Overtime, Long work hours, Cardiovascular disease, Biomarkers

## Abstract

Shift work and overtime have been implicated as important work-related risk factors for cardiovascular disease (CVD). Many firefighters who contractually work on a 24-hr work schedule, often do overtime (additional 24-hr shifts) which can result in working multiple, consecutive 24-hr shifts. Very little research has been conducted on firefighters at work that examines the impact of performing consecutive 24-hr shifts on cardiovascular physiology. Also, there have been no standard field methods for assessing in firefighters the cardiovascular changes that result from 24-hr shifts, what we call “cardiovascular strain”. The objective of this study, as the first step toward elucidating the role of very long (> 48 hrs) shifts in the development of CVD in firefighters, is to develop and describe a theoretical framework for studying cardiovascular strain in firefighters on very long shifts (i.e., > 2 consecutive 24-hr shifts). The developed theoretical framework was built on an extensive literature review, our recently completed studies with firefighters in Southern California, e-mail and discussions with several firefighters on their experiences of consecutive shifts, and our recently conducted feasibility study in a small group of firefighters of several ambulatory cardiovascular strain biomarkers (heart rate, heart rate variability, blood pressure, salivary cortisol, and salivary C-reactive protein). The theoretical framework developed in this study will facilitate future field studies on consecutive 24-hr shifts and cardiovascular health in firefighters. Also it will increase our understanding of the mechanisms by which shift work or long work hours can affect CVD, particularly through CVD biological risk factors, and thereby inform policy about sustainable work and rest schedules for firefighters.

## Introduction

There are almost 1.2 million professional and voluntary firefighters in the United States (US) [1]. Firefighters have a high risk for on-duty cardiovascular disease (CVD) mortality [2,3] and have a high prevalence of CVD biological risk factors (obesity, hypertension, and hyperlipidemia) [2-6]. Working consecutive 24-hr shifts, a combination of 24-hr shift work and overtime (another 24-hr shifts), is common [4,6,7] among US firefighters. However, very little research has been conducted on firefighters at work that examines the impact of performing consecutive 24-hr shifts on their cardiovascular physiology. Also there have been few established physiological methods for assessing cardiovascular strain in firefighter on consecutive 24-hr shifts. The objective of this study, as the first step toward elucidating the role of very long (> 48 hrs) shifts in the development of CVD in firefighters, is to develop and describe a theoretical framework for studying cardiovascular strain in firefighters on very long shifts (i.e., > 2 consecutive 24-hr shifts).

## Review

### Consecutive 24-hr Shifts among Firefighters

According to the 2010 firefighter fatality report of the US Fire Administration, sudden cardiac death accounts for 49% of total firefighter fatalities at work. The reasons for these increased fatalities are not well understood and we believe there may be important unidentified or understudied occupational determinants of CVD risk factors contributing to CVD mortality in firefighters. Shift work and overtime (additional 24-hr shifts) are very common among firefighters in the US [4,6,7] and have been implicated as important work-related risk factors of CVD in firefighters [5,8] based on the literature on shift work or long work hours and CVD in general or among other working populations.

Firefighters usually work a 24-hr shift schedule (e.g., the Kelly schedule: On/Off/On/Off/On/Off/Off/Off/Off; a modified Kelly schedule; or a 48-hr work on and 96-hr off schedule: On/On/Off/Off/Off/Off). However, many firefighters do additional 24-hr shifts beyond their standard work schedule (10-11 shifts per month) (for example, see Table 1). Overtime can be voluntary or mandatory, and is due to a constant staffing policy – “staffing an operation with just enough positions to cover all seats – leaves and vacancies are covered with overtime assignments” [9]. This policy frequently results in firefighters working multiple consecutive 24-hr work shifts per month. A recent nationwide survey on firefighter departmental regulations regarding consecutive work hour limits among 37 fire departments in the US [7] indicated that the majority of fire departments (62%) allowed firefighters to do shifts of 72 or more consecutive work hours: many have no limit to the number of consecutive shifts (35%); while some have a 72 work hour limit (24%); or a 96 work hour limit (3%). In our recent survey of 365 firefighters [4,10] who were employed at a fire department in Southern California, firefighters worked on average thirteen 24-hr shifts per month which is 2–3 more shifts per month than required by contract. Moreover, a substantial number of firefighters, 67%, worked 72 consecutive hours, while 26% worked 96 consecutive hours at least one time per month. On a typical 24-hr shift, firefighters can sleep at night at the fire station, but they can be frequently woken for emergency calls.

**Table 1:** A 24-hr work schedule of the three (ABC) crews of firefighters in Southern California in a month of 2012

**Day**	**1**	**2**	**3**	**4**	**5**	**6**	**7**	**8**	**9**	**10**	**11**	**12**	**13**	**14**	**15**	**16**	**17**	**18**	**19**	**20**	**21**	**22**	**23**	**24**	**25**	**26**	**27**	**28**	**29**	**30**	**31**	**Total**
A crew	-	O	-	-	O	-	O	-	-	-	-	O	-	O	-	-	O	-	O	-	-	-	-	O	-	O	-	-	O	-	O	11 shifts
B crew	O	-	O	-	-	-	-	O	-	O	-	-	O	-	O	-	-	-	-	O	-	O	-	-	O	-	O	-	-	-	-	10 shifts
C crew	-	-	-	O	-	O	-	-	O	-	O	-	-	-	-	O	-	O	-	-	O	-	O	-	-	-	-	O	-	O	-	10 shifts

O: on-duty 24-hr work day, −: off-duty day.

A number of occupations already have nationwide regulations on work and rest schedules, such as drivers (continuous driving time, ≤10 hrs) [11], young doctors (Accreditation Council for Graduate Medical Education, continuous working time, ≤ 30 hrs) [12], and ambulance workers (American Ambulance Association, continuous working time, ≤ 36 hrs) [7]. But firefighters do not have one, in part, because there is as yet insufficient evidence of the potential harm of such work schedules for firefighters.

### Little is known about the impact of consecutive 24-hr shifts on the health of firefighters

Despite numerous epidemiological studies [13-15] on the causes of CVD-related mortality in firefighters, no studies have specifically examined the impact of consecutive 24-hr shifts on CVD or CVD biological risk factors in firefighters. One study [16] reported that sick leave, work-related injury, and motor vehicle accident rates were higher among firefighters on the 2nd 24-hr shift than on the 1st 24-hr shift. Only two studies [17,18] examined the association between 24-hr shifts and mental health among firefighters. Saijo et al. [17] reported that there was no significant association between the number of 24-hr shifts per month (11–13 times vs. 8–10 times) and depression symptoms in 1,301 firefighters. However, the authors reported higher (albeit not statistically significant) depression symptoms in firefighters who reported longer extra hours per month (4–33 hrs vs. 0–3 hrs). In our study, we found higher self-reported exhaustion on the Maslach Burnout Inventory [19] among firefighters in Southern California doing frequent three consecutive 24-hr shifts per month [18]. Depression and exhaustion have been suggested as possible CVD risk factors [20,21] that could disturb several internal physiological systems (e.g., autonomic nervous system, hypothalamus-pituitary-adrenal (HPA) axis, and systematic inflammation process).

Three NIOSH firefighter fatality reports [22-24] investigated sudden death cases which occurred in the milieu of consecutive 24-hr shifts and NIOSH proposed to limit the number of consecutive shifts that a firefighter can work for preventing on-duty sudden cardiac death among firefighters based on the literature in other working populations that shift work and long work hours may impact on CVD [25,26]. Although one may infer from the above studies that consecutive 24-hr shifts increase the risk of CVD among firefighters, there is as yet no strong evidence that multiple consecutives 24-hr shifts are associated with CVD risk factors or with cardiovascular strain and CVD in firefighters.

### Two critical barriers to progress in research on cardiovascular strain in firefighters on very long shifts

One critical barrier to progress in research on cardiovascular strain in firefighters is the lack of a well-designed field study that examines day-to-day changes in cardiovascular parameters in firefighters working consecutive shifts. Conduct of such a field study has been recommended to determine whether there is an association between long work hours and health-related outcomes [27] mediated by cardiovascular mechanisms. Also it is well known that a within-subject study design, i.e., repeated measures in a subject over time, has merits over a between-subject study in that it has more statistical power (smaller sample size needed for study) and is more effective in controlling for individual differences in coping and physiological responses to environmental stressors [27-29] because the participants act as their own control group.

Another critical barrier to progress is the lack of standard methods for assessing the accumulation of cardiovascular strain in firefighters over consecutive 24-hr shifts. Strain is defined here as the internal responses of the organisms to external stimuli (e.g. work stressors) in accordance with the classical stress–strain workload model [30,31]. More specifically, cardiovascular strain is defined as the physiological responses of the cardiovascular system (heart and blood vessels) to external stimulus. It can be detected by measuring the stimulus-induced changes in a number of physiological parameters related to the control of the cardiovascular system, either directly (e.g., heart rate and blood pressure) or indirectly (e.g., cortisol [32] and C-reactive protein (CRP) [33]. Heart rate [34-38], heart rate variability [39,40], and blood pressure [41-43] are well-known CVD risk factors that have been used for decades in occupational ergonomics for assessing cardiovascular strain resulting from dynamic physical work [30] or mental workload [29]. Heart rate and blood pressure are also linearly associated with CV events [36,44]. However, it has never been fully tested whether those and other promising measures (e.g., heart rate variability or salivary cortisol) can be used as “sub-clinical” biomarkers to assess cardiovascular strain accumulating over consecutive 24-hr shifts in firefighters. A few physiological studies [45-48] have shown a significant increase in heart rate when a firefighter responds to a call and also demonstrated a significant increase in heart rate variability when a favorable (fatigue-reducing) work schedule was introduced at night, but none of them extended their assessment beyond one 24-hr shift for the purpose of measuring the impact of consecutive shifts in firefighters. Also, no standard tests are available for assessing cardiovascular strain in firefighters in the wellness and fitness (WEFIT) medical programs implemented by many fire departments in the US (for details, http://www.iaff.org/HS/Well/wellness.html). The treadmill or cycle ergometer test in WEFIT medical programs is designed to estimate the maximal aerobic power (VO_2_ max) of firefighters and it is a good indicator of the physical working capacity or fitness of a firefighter. But it is limited in predicting or estimating cardiovascular strain in firefighters. About 30-40% of the VO_2_max of a worker has been used as an acceptable physical workload in occupational ergonomics [49-51]; however, the cut-point of 30-40% of VO_2_max is determined based on the assumption that a worker does physical tasks for 8 hours a day. Wu et al. [51] demonstrated that “long-hour shifts (> 10 hr) should assign a lower work intensity than for an 8-hr workday”. In addition, firefighters are not only exposed to variable amounts of physical work demands, but also numerous psychosocial mental demands at work [4,5], so their total workload also cannot be determined based on a physical workload measure alone.

### A feasibility study with several ambulatory biomarkers of cardiovascular strain

We recently recruited a group of firefighters (N = 7) in Southern California for a feasibility study of measuring several cardiovascular strain biomarkers (heart rate, heart rate variability, blood pressure, saliva cortisol, and saliva CRP) while they were doing 3 consecutive 24-hr shifts. We used recent, technologically-advanced, non-invasive instruments (a Polar S810 heart rate monitor, an Omron HEM-670 wrist blood pressure monitor, and Salimetric salivary cortisol and CRP test kits) for the data collection of the biomarkers and a short diary for the data collection of self-reported fatigue and distress. The data were collected at fire stations on the 1st and 3rd (not 2nd shift for simplicity) 24-shifts during 3 consecutive 24-hr shifts being completed by each of seven firefighters. The protocol of the feasibility study was reviewed and approved by the Institutional Review Board of the University of California, Irvine (HS# 2011-8426).

We chose five key physiological parameters that should be examined as “sub-clinical” biomarkers to assess cardiovascular strain accumulating over consecutives shifts in firefighters. The decision was made based on 1) the relevance of the parameters to CVD: all of the 5 parameters - heart rate [34-38], heart rate variability [39,40], blood pressure [41-43], cortisol [52,53], and C-reactive protein [54] have been reported as important CVD risk factors, and 2) the diverse physiological functions that the parameters indicate, considering the physiological mechanisms by which consecutive shifts could affect CVD. The Polar S810 heart rate monitor can measure not only heart rate, but also heart rate (beat-to-beat) variability with a resolution of 1 ms [55]. It is less expensive and simpler (no shaving, easier in detaching and reattaching when needed, and auto HR and HRV artifact correction functions) compared to a traditional ambulatory electrocardiogram (ECG) monitor (a Polar Holter monitor). The Polar S810 heart rate monitor was validated against an ECG monitors by several investigators [55,56]. Wrist blood pressure monitors with advanced positing sensor (APS) have been validated against mercury sphygmomanometers according to the International Protocol of the European Society of Hypertension [57,58].

In our feasibility test, firefighters successfully assessed their blood pressure with the Omron wrist blood pressure monitor every hour during the daytime at the fire station on consecutive 24-hr shifts. Saliva cortisol has been widely used for decades due to its simple and non-invasive characteristics [59]. Saliva CRP has not been used widely because of the lack of information about the relationship between saliva CRP and serum CRP. However, recent papers reported that there is a high correlation between serum and saliva CRP levels [60] and a significant difference in salivary cortisol between healthy and cardiac patients [61] and between non-smokers and smokers [62]. Saliva CRP is a promising non-invasive CVD biomarker that can be easily collected and analyzed along with salivary cortisol, although its relationship with work demands has not been explored in firefighters and other occupations. Our pilot study supported the feasibility of the assessment of several cardiovascular strain biomarkers and self-rated stress and fatigue while firefighters were doing a very long shift.

### Developing a theoretical framework for studying cardiovascular strain in firefighters on very long shifts

Researchers [63-65] have proposed several mechanisms whereby shift-work could affect CVD. These include the possibility that shift work may increase the risk of CVD by 1) disturbance of circadian rhythms of physiological parameters that are related to the cardiovascular system, 2) stress responses from the exposure to adverse psychosocial working conditions (e.g., job strain or effort-reward imbalance) including disrupting normal social relations (e.g., with family), and/or 3) changes in health behaviors (e.g., sleep quality, eating behaviors, and physical activities). In addition, long working hours (overtime) can aggravate the aforementioned effects of shift work and may significantly increase the risk of CVD when combined with decreased recovery, particularly insufficient sleep [27,66]. Although the proposed mechanisms are a good general guide for research on long-term effects of shift work and long work hours and CVD, they need to be further elucidated and specified for research in firefighters.

**Figure 1:**
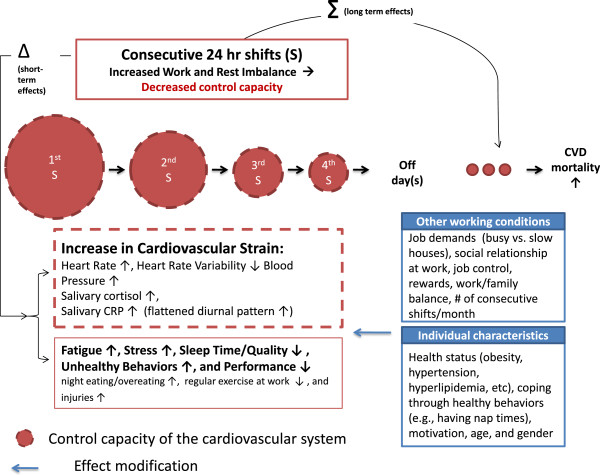
A theoretical framework for studying the impact of consecutive 24-hr shifts on the cardiovascular system in firefighters.

We developed a theoretical framework for studying cardiovascular strain in firefighters on consecutive 24-hr shifts. The developed theoretical framework was built on an extensive literature review, our recently completed studies with firefighters in Southern California, e-mail and discussions with several firefighters on their experiences of consecutive shifts, and our recently conducted feasibility study of several ambulatory cardiovascular strain biomarkers in a small group of firefighters. We suppose that a firefighter has his/her own capacity (“the maximum capacity of a person”) [67] to perform given tasks while maintaining internal psychological and physiological homeostasis [68,69] (hereafter called, “control capacity” in Figure 1). It is consistent with contemporary ergonomic and systems biology models, for example, the resource model [70] in which workload is defined as the proportion of the maximum capacity used to do given tasks and also with the stress-disequilibrium theory [71] built on an application of systems theory [72] into biology, in which physiological ordering capacity was defined as “a limit on the ability of the organism to internally organize its adaptive interactions with its environments”.

Based on the “heuristic” concept, we hypothesize that the control capacity of the cardiovascular system in firefighters will decrease to some extent through a single 24-hour shift, but it will return to its initial level of capacity following 24-hr off-duty days. However, if a firefighter does 24-hr shifts consecutively, particularly 72 or 96 hours of consecutive work, the control capacity of the cardiovascular system in the firefighter will decrease significantly due to increased work and rest imbalance, which results in accumulating cardiovascular strain over consecutive 24-hr shifts (see Figure 1). Particularly, several interrelated physiological systems or processes such as the autonomic nervous system, hypothalamus pituitary adrenal (HPA) axis, baroreceptor reflex dysfunction, and inflammation processes, all of which are involved in the control of the cardiovascular system will be disturbed at the end of the consecutive shifts. The over-activation of the sympathetic nervous system will result in increased heart rate and blood pressure. The autonomic imbalance will lead to a decrease in heart rate (beat-to-beat R-R interval) variability. Over-activation of the HPA axis will increase salivary cortisol level, particularly in the early morning (usually salivary cortisol increases rapidly after awakening and has its highest value about 30 minutes just after awakening). Increased systematic inflammation may result in an increase and/or a flattened diurnal pattern of salivary CRP. CRP has the highest value just after awakening and the lowest value at midday [73]. If cardiovascular strain accumulates over years through repeated multiple consecutive 24-hr shifts (particularly 72 or 96 hours of consecutive work), it will lead to the “irreversible” loss in control capacity of the cardiovascular system in firefighters (marked as a red real line of the circle in Figure 1 vs. reversible loss marked as a dotted line).

We think that consecutive 24-hr shifts are likely to contribute to psychological strain (e.g., fatigue and distress), sleep disturbance, unhealthy behaviors, and decreased performance at work among firefighters in line with existing literature on long work hours and shift work [25,74,75]. It is well-known that physiological parameters (e.g. heart rate, blood pressure, cortisol, and CRP) are influenced by psychological strain, sleep, and health behaviors and that vice versa is true [67,76-78]. For instance, one experimental study [77] in 10 healthy young adults reported that when they did not have any sleep for 3 days their systolic and diastolic blood pressure and pulse rate dramatically increased from Day 1 to Day 3. High cortisol reactors to a given stressor consumed more calories compared to low cortisol reactors [79]. Nonetheless, one issue regarding blood pressure needs to be discussed for clarity. Some studies have reported that exhaustion [21] and fatigue [80] are negatively associated with blood pressure (that is, the fatigued have lower blood pressure compared to the non-fatigued). However, we think, as Stewart and Write et al. [81] pointed out, that the effect of exhaustion or fatigue on blood pressure at least partly depends on whether the fatigued are willing to or are forced to make more effort given their fatigued status. We think that firefighters cannot and do not give up their social role as first emergency respondents even when they are fatigued. So we anticipate that fatigued firefighters will show higher blood pressure rather than lower blood pressure compared to non-fatigued firefighters.

We think that the cardiovascular strain that results from working very long shifts will differ by several working conditions and individual characteristics. We hypothesize that cardiovascular strain from very long shifts will be higher among firefighters at a busy fire station since they will have less recovery time during the day or at night due to a higher volume of calls. However, we do not exclude the possibility that firefighters at slow stations could also be internally strained for a long period of time because they have to be ready for any unpredictable calls. We think that other adverse psychosocial working conditions (e.g., job strain, effort-reward imbalance, poor social relationships at work, work and family conflict, and frequent consecutive shifts per month) will also increase cardiovascular strain directly through stress [82-84] or indirectly through changes in health behaviors [85].

Firefighters who already have traditional CVD risk factors (e.g., obesity, hypertension, and hyperlipidemia) may experience higher cardiovascular strain during consecutive 24-hr shifts, compared to healthy firefighters without CVD risk factors. It has been reported that increased resting heart rate is associated with obesity [86,87] and high triglyceride and cholesterol [88] and low heart rate variability is associated with obesity [89], hypertension [90], and hyperlipidemia [91]. However, there is little information on any differential reactivity to a given workload by health status. One study [92] among air-traffic controllers reported that a higher cardiovascular reactivity to workload (defined as the number of planes on the air traffic control screen) was a significant predictor of hypertension 20 years later. This implies that cardiovascular reactivity to a given task may be higher in hypertensive workers. However, this was not the case in one Japanese study [93] in which the blood pressure reactivity to a long work schedule (60 hrs per week) among mild-hypertensive workers was slightly less (1 mm Hg) than among normotensive workers. But in the same study [93], there was a higher reactivity of 24-hr heart rate to the long work schedule among hypertensive workers (5 beats per min vs. 3 beats per min).

A recent experimental animal study [94] reported that obese rats showed a higher cardiovascular reactivity (of heart rate and blood pressure) to the stress of immobilization than non-obese rats. We think (based on e-mail discussions with firefighters in Southern California) that some firefighters try to reduce stress from consecutive 24-hr shifts by increasing healthy behaviors (e.g., exercising at work, eating healthy foods, and having nap times during the day). Thus there is a possibility that firefighters and fire departments can reduce cardiovascular strain in firefighters on consecutive 24-hr shifts by promoting the healthy behaviors of individual firefighters and improving fire department-level safety and health climates which encourage healthy behaviors.

Finally, it is possible that the impact of consecutive shifts on the cardiovascular system of firefighters will differ by the motivation of firefighters (financial needs) and the context of the consecutive shifts (whether they choose the consecutive shifts or are forced to work them; “mandatory vs. voluntary” overtime). On the other hand, the high financial rewards received for working additional shifts may result in decreased cardiovascular strain.

## Conclusions

Despite the fact that very long (> 48 hrs) shifts are common among firefighters in the United States, little is known about the impact of consecutive 24-hr shifts on the cardiovascular health of firefighters. As the first step toward our long-term goal of elucidating the role of very long shifts in the development of CVD in firefighters, we developed and described a theoretical framework for studying cardiovascular strain in firefighters on very long shifts (i.e., 3 or 4 consecutive 24-hr shifts) based on an extensive literature review and several recent studies with firefighters in Southern California. We think the theoretical framework developed in this article will facilitate future field studies on consecutive 24-hr shifts and cardiovascular health in firefighters and increase our understanding of the mechanisms by which shift work or long work hours can affect CVD, particularly through CVD biological risk factors.

To the best of our knowledge, few studies have been conducted to examine the associations between very long work shifts and CVD or CVD biological risk factors [25], although some studies have examined the risks of very long shifts for injury and fatigue [95]. Also several researchers [74,75] have recently emphasized the importance of understanding the mechanisms in order to interpret the epidemiological studies on shift work and CVD correctly. A well-designed field study based on the theoretical framework and a within-subject study design in firefighters is urgently needed in the near future. The future study will meet the public safety sub-sector strategic goal 1.5 (particularly, 1.5.3: “investigate biological mechanisms between psychosocial and physical stressors and subclinical markers of cardiovascular disease”) of the National Occupational Research Agenda (NORA) at the National Institute for Occupational Safety and Health (NIOSH).

Lastly, we think the theoretical framework developed in this study could ultimately contribute to establishing a nationwide guideline about sustainable 24-hr work and rest schedules for firefighters with which a firefighter can work without a significant burden on his/her cardiovascular system.

## Consent (adult)

A written informed consent was obtained from each of the firefighters who participated in the aforementioned studies.

## Competing interests

All authors have no competing interests to declare.

## Authors’ contributions

BC conceived and designed this study. PS, MD, HK, LI, and DB contributed to the design and the literature review of this study. JG contributed to the data collection at fire stations. FZ contributed to the data analysis of saliva samples from firefighters at fire stations. BC drafted this manuscript. All authors (particularly PS, MD, and DB) revised this manuscript critically. All authors read and approved the final manuscript.
